# Current practices in obstetrics and gynecology transition to residency courses: graduates’ perspectives on activities, topics, and overall educational benefit

**DOI:** 10.1186/s12909-026-08977-3

**Published:** 2026-06-12

**Authors:** Haley Fox, Cynthia Garvan, Archana Pradhan, Shireen Madani Sims, Bradley Bruggeman

**Affiliations:** 1 Department of General Surgery, University of Florida College of Medicine, 1600 SW Archer Rd, Gainesville, Fl 32610 USA; 2 Department of Obstetrics and Gynecology, University of Florida College of Medicine, 1600 SW Archer Rd, Gainesville, FL 32610 USA; 3 Department of Obstetrics and Gynecology, Rutgers Robert Wood Johnson Medical School, 125 Paterson St, New Brunswick, NJ 80901 USA

**Keywords:** OBGYN, Transition to Residency, TTR, Bootcamp, Fourth year, Residency

## Abstract

**Background:**

Transition to residency (TTR) courses are increasingly implemented in U.S. medical schools and result in increased confidence among incoming residents (Acad Med J Assoc Am Med Coll 90:1324-1330, 2015, Acad Med 90:1116, 2015). There is limited literature describing TTR courses in Obstetrics and Gynecology (OBGYN). This study aimed to characterize the structure, content, and perceived benefit of OBGYN TTR courses.

**Methods:**

An anonymous observational cross-sectional survey was distributed to all residents at 22 OBGYN residency programs between July and September 2024. The 36-item electronic questionnaire assessed residents’ participation in an OBGYN TTR course, and if so, its duration, structure, activities, topics, and use of competency-based assessments. Respondents rated the course and individual activities and topics using a 5-point Likert scale and identified the most important topics to include in a TTR course. Descriptive statistics and Kruskal–Wallis tests were used for analysis.

**Results:**

A total 121 of 552 residents (21.9%) responded to the survey, from the 22 programs to which the survey was sent. Residents represented 73 medical schools from all U.S. census regions. Nearly half of respondents (45.5%) reported receiving no OBGYN TTR course. Most were integrated within a general TTR course and spanned two weeks or less. The most common learning activities were didactic lectures (85%) and surgical skills lab (68%) with residents favoring ambulatory procedure, surgical, and ultrasound skills labs and obstetric simulations. There was a large overlap between topics residents perceived most important to include and those that are currently most utilized. These topics included suturing and knot tying (82%), normal obstetric care (40%), abdominal/pelvic anatomy (40%), and intrapartum care and conduct of normal labor (37%). Few respondents received competency-based assessments such as APGO’s PrepForRes quiz (19.1%) or a standardized handover (3.1%).

**Conclusions:**

OBGYN TTR courses are inconsistently implemented across U.S. medical schools. Residents perceive greater educational value from experiential learning and from core clinical topics. These findings highlight opportunities to expand and standardize OBGYN TTR curricula, emphasize hands-on learning modalities, and incorporate milestone-based assessments to better prepare graduates for the transition to residency.

**Supplementary Information:**

The online version contains supplementary material available at 10.1186/s12909-026-08977-3.

## Background

Over the past two decades, increasing attention and scholarship [1] has been devoted to optimizing curriculum in the fourth year of medical school, particularly in preparing students for the transition to residency. The Alliance for Clinical Education published recommendations for redesigning the fourth year in 2014, including competency-based transition-to-residency (TTR) “capstone courses” or “bootcamps” with general and specialty-specific recommendations. A 2015 review [2] reported that such courses were required by 59% of medical schools – a sharp contrast compared to a 2001 comprehensive survey [3] of medical school curricular requirements which made no mention of TTR courses. Studies have shown that TTR courses produce short-term advancement in medical knowledge, procedural skills, and overall performance with the most common benefit being an increased confidence upon entering intern year [2]. This increased self-assurance early in residency is valuable because OBGYN residents have reported lack of preparedness in specific skills (e.g. ultrasound, suturing) and in medical knowledge of routine inpatient issues upon entering residency [4]. There is considerable variability in the structure and content of TTR courses, though most are focused on general “day-one readiness” skills including organization and time management, effective communication, hospital/team function, and basic clinical skills relevant to the intern [2].

The Coalition for Physician Accountability’s Undergraduate Medical Education-Graduate Medical Education Review Committee (UGRC) has recommended that specialty-specific training be provided to all incoming first-year residents. In 2013, the Accreditation Council for Graduate Medical Education (ACGME) adopted Milestones, which are competency-based developmental outcome expectations that can be demonstrated progressively by residents from the first postgraduate year (PGY1) through graduation. The UGRC recommendation, coupled with the benchmark that students achieve Milestone Level 1 prior to entering PGY1 year, provides an excellent opportunity for TTR curricular design that is competency-based and specialty-specific. The majority of literature [1] in specialty-specific TTR courses has been in general surgery and surgical subspecialties. In fact, the American College of Surgeons developed a national standardized TTR curriculum that was successfully piloted at 93 U.S. medical schools [5]. One study showed that surgical interns who received a surgery-specific TTR course reported higher perceived competency in surgical technical skills, professionalism, interpersonal communication skills, and overall preparation compared to interns who did not [6].

A recent scoping review [1] reported a significant increase in the use of competency-based medical education (CBME) in the final year of medical school. However, while 74% of published TTR courses reference a CBME framework in their design, this does not directly translate to the implementation of adequate structured assessments for learner competency.

Within the field of obstetrics and gynecology (OBGYN), there is a relative paucity of literature regarding TTR courses, both in terms of content and structure. Morgan et al. published [7] a single institution’s pilot TTR curriculum based on OBGYN-specific Milestones Level 1 in 2014 and subsequently demonstrated [8] improvement in medical knowledge assessments that were sustained to the start of PGY1 year. The literature appears to lack any other published institution-specific curricula. Accordingly, little is known regarding the current state of OBGYN TTR courses nationwide, such as prevalence, duration, content covered, educator and learner perceptions of the value of various activities, and assessment. The Association of Professors of Gynecology and Obstetrics (APGO) has developed self-directed learning [9, 10] and self-assessment [11] resources over the past five years, though the extent to which these are implemented is limited.

To address these gaps, a survey of current OBGYN residents was conducted regarding their experiences with fourth-year medical school OBGYN TTR courses. This study aimed to (1) describe the prevalence and structure of existing OBGYN TTR courses, (2) characterize learners’ perceptions of helpfulness, and (3) identify content areas of highest perceived educational value. This study is the first national survey assessing OBGYN TTR courses from residents’ perspectives.

## Methods

### Resident survey and participants

An observational cross-sectional survey of residents from 22 U.S. OBGYN residency programs was performed using a 36-item electronic survey (Appendix A) that was designed for this study. Previously-published studies and ACGME Milestones were used to inform the design and content of questionnaire items [4, 6, 7, 12]. Demographic information included current level of residency training, degree type, and geographic location of their medical school. Respondents indicated whether they participated in an OBGYN TTR course, and if so, whether it was stand-alone or nested within a general TTR course and its duration, content, methods, and perceived helpfulness. Respondents were also asked if they completed competency-based assessments.

Respondents indicated whether they received education about 19 topics that were mapped to ACGME Patient Care Milestones Level 1 for OBGYN residents (Appendix B) to reflect established competencies. Respondents also indicated which educational activities were included in their TTR course. These activities were selected for inclusion based on previously published TTR curricula and needs assessments [4]. The perceived degree of helpfulness of the TTR course overall, as well as the educational topics and activities described above, were each assessed by a 5-point Likert scale (1 = “not at all helpful” to 5 = “extremely helpful”). Finally, all respondents were asked to indicate which five Milestone Level 1- mapped topics from a provided list would have been most helpful to prepare them for the start of residency.

Regarding assessment, respondents were asked about completion of APGO’s Preparation for Residency Knowledge Assessment Tool (PrepForRes quiz) and a standardized handover. The PrepForRes quiz is a 100-item multiple-choice assessment mapped to ACGME Milestones Level 1 that was developed and is continuously updated using psychometric data by the leading body of academic obstetrician-gynecologists and is encouraged for completion by all incoming OBGYN residents [11]. A handover is a report completed at the learner’s medical school that outlines competencies for their new residency program director.

The survey was pilot tested for clarity and time in a focus group comprising 18 residents from a single institution. These survey responses were excluded from final analysis. Based on focus group feedback, explanatory sentences were included to clarify questions with pedagogical terms such as “handover” and “flipped classroom”, and examples were provided to clarify some educational activity types such as “surgical skills labs” and “ambulatory procedures labs”.

We used a convenience sample of programs affiliated with a cohort of collaborators from the APGO’s Academic Scholars and Leaders program. All OBGYN residents at 22 of the 291 U.S. OBGYN residency programs in 2024 [13] (7.6%) were invited to complete the survey (Appendix C). The programs were broadly distributed across geographic regions and represented diverse program settings (university-based and community-based, university affiliated). Invitation to complete the survey was delivered via email in July 2024 (*n* = 552). Follow-up emails were sent four weeks later in August 2024 and the survey was closed in September 2024. Participation was voluntary and anonymous, and there were no incentives for participation.

### Statistical analysis

Data were checked for implausible values and distributional form, then summarized using percentages, medians, interquartile ranges, and visual displays. The approach for handling missing data in all analyses was pairwise deletion (i.e., available-case analysis). Likert scale ordinal data were compared among groups using Kruskal–Wallis tests. The level of significance was set at 0.05 and all hypothesis testing was two-sided. SAS version 9.4 (Cary, NC) was used for all data analysis.

## Results

### Respondent characteristics

A total of 121 of the 552 residents who were contacted completed the survey and were included in the survey analysis, for a response rate of 21.9%. Residents represented graduates from 73 of the 193 U.S. medical schools (38%) accredited by the Liaison Committee for Medical Education (LCME). Medical schools of all U.S. census regions were represented.

Graduates from MD granting institutions were over-represented with 81% of the survey responses, compared to MD graduates filling 71.7–74.5% of the OBGYN PGY-1 match positions 2021–2024 [13–16]. Respondents represented all years of residency training, although with an expected national average of 25% per PGY class, PGY1 residents were over-represented (34.7%) and PGY4 residents were under-represented (17.4%). Table 1 describes the demographics of respondents, including year of training, degree received by medical school (MD or DO), and U.S. census regions of their medical school.

**Table 1:** Demographics of survey respondents

Survey Responses (*N* = 121)	N (%)
Current level of training	
PGY1	42 (34.7)
PGY2	28 (23.1)
PGY3	30 (24.8)
PGY4	21 (17.4)
Undergraduate institution type	
U.S. MD granting institution	98 (81.0)
U.S. DO granting institution	23 (19.0)
U.S. census region of medical school	
New England (ME, NH, VT, MA, RI, CT)	7 (5.8)
Middle Atlantic (NY, NJ, PA)	19 (15.7)
East North Central (OH, MI, IN, IL, WI)	19 (15.7)
West North Central (MN, ND, SD, IA, NE, MO, KS)	10 (8.3)
South Atlantic (DE, MD, DC, WV, VA, NC, SC, GA, FL)	40 (33.1)
East South Central (KY, TN, AL, MS)	5 (4.1)
West South Central (AR, OK, LA, TX)	3 (2.5)
Mountain (MT, ID, WY, CO, UT, NV, NM, AZ)	6 (5.0)
Pacific (AK, WA, OR, CA, HI)	12 (9.9)

Postgraduate year (PGY), United States (U.S.), Doctor of Medicine (MD), Doctor of Osteopathic Medicine (DO), Maine (ME), New Hampshire (NH), Vermont (VT), Massachusetts (MA), Rhode Island (RI), Connecticut (CT), New York (NY), New Jersey (NJ), Pennsylvania (PA), Ohio (OH), Michigan (MI), Indiana (IN), Illinois (IL), Wisconsin (WI), Minnesota (MN), North Dakota (ND), South Dakota (SD), Iowa (IA), Nebraska (NE), Missouri (MO), Kansas (KS), Delaware (DE), Maryland (MD), District of Columbia (DC), West Virginia (WV), Virginia (VA), North Carolina (NC), South Carolina (SC), Georgia (GA), Florida (FL), Kentucky (KY), Tennessee (TN), Alabama (AL), Mississippi (MS), Arkansas (AR), Oklahoma (OK), Louisiana (LA), Texas (TX), Montana (MT), Idaho (ID), Wyoming (WY), Colorado (CO), Utah (UT), Nevada (NV), New Mexico (NM), Arizona (AZ), Alaska (AK), Washington (WA), Oregon (OR), California (CA), Hawaii (HI)

### TTR course helpfulness, duration, and assessment

**Fig. 1:**
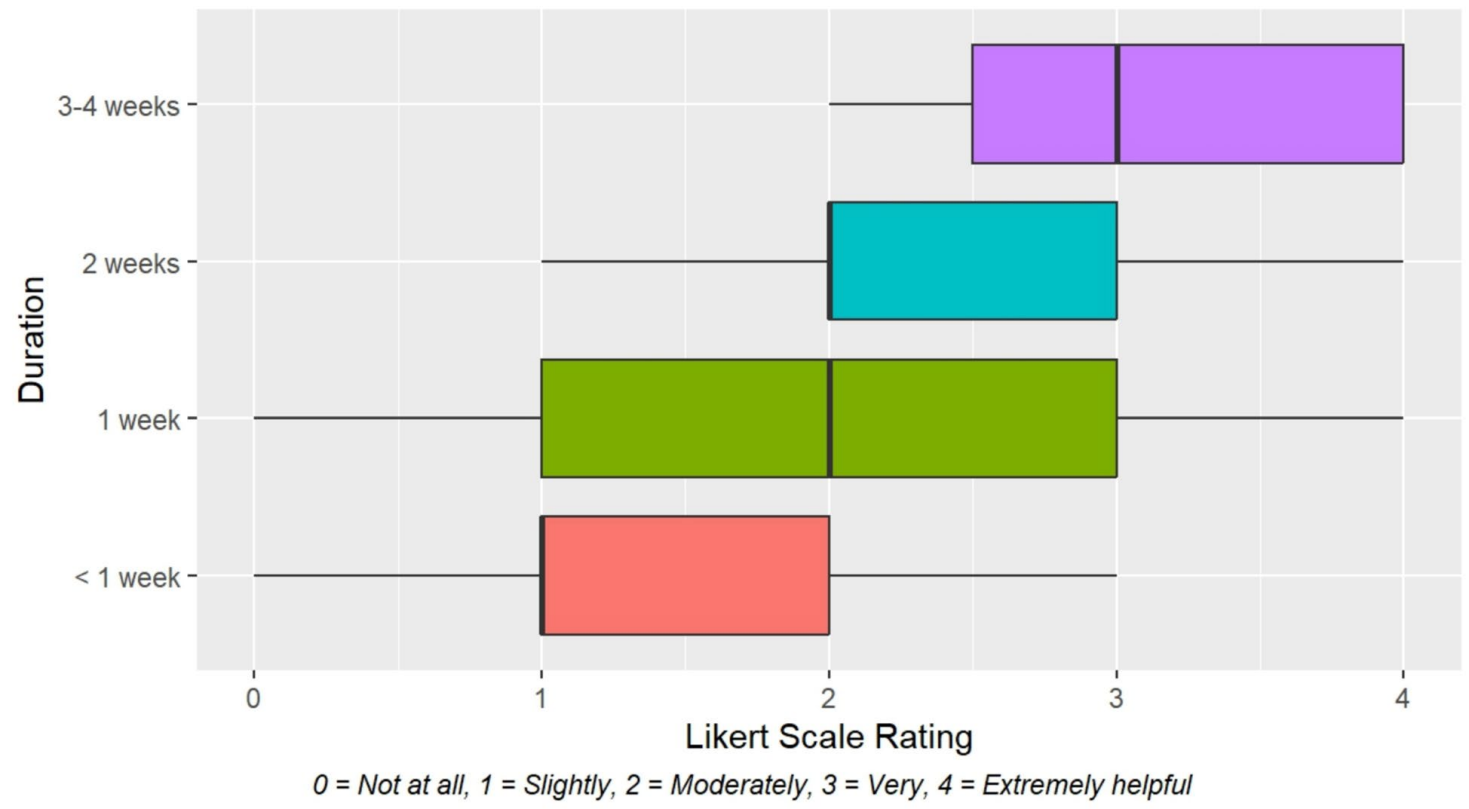
Perceived helpfulness of OBGYN TTR course across duration of course. Respondents who received an OBGYN TTR course were asked how helpful the course was on a Likert scale and how long the course content was from < 1 week to 4 weeks. (*n* = 64 respondents who received an OBGYN TTR course)

**Fig. 2:**
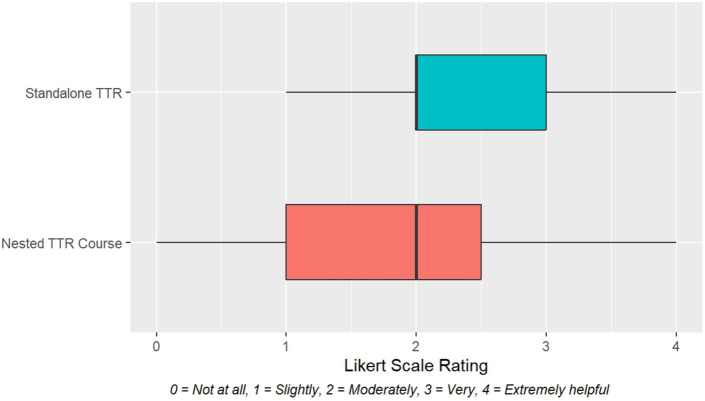
Perceived helpfulness of OBGYN TTR course across course structure. Respondents who received an OBGYN TTR course were asked how helpful the course was on a Likert scale and whether the content was delivered as a stand-alone course or whether it was integrated into a general TTR course. (*n* = 64 respondents who received an OBGYN TTR course)

Table 2 summarizes the presence of and general composition of OBGYN TTR courses and assessments. Nearly half (45.5%) of respondents received no OBGYN content in a TTR course. Most respondents who received an OBGYN TTR course (68.1%) reported that this content was nested within a general TTR capstone course. Most respondents (62.5%) regarded the course as at least moderately helpful. With duration ranging from “ < 1 week” to “4 weeks”, most respondents (88.8%) received two weeks or less of OBGYN-focused content. We found that respondents’ perception of helpfulness was positively associated with a longer duration of the course and with a standalone structure (*p* = 0.0026, *p* = 0.0230 respectively, Kruskal–Wallis tests). Boxplots of helpfulness scores for various course durations and course structures are depicted in Figs. 1 and 2, respectively. Regarding objective assessments of competency, few respondents reported completing APGO’s PrepForRes quiz (19.1%) or receiving a competency handover to share with their program director (3.1%).

**Table 2:** General aspects of OBGYN transition to residency courses

Survey Responses	
Did you complete an OBGYN Transition to Residency “bootcamp” at the end of your fourth year of medical school? (*N* = 121)	N (%)
Yes – I took a stand-alone OBGYN bootcamp elective	21 (17.4)
Yes – OBGYN content was integrated into a general transition to residency bootcamp	45 (37.2)
No – I did not receive OBGYN specific residency preparatory training	55 (45.5)
How long was the OBGYN-specific training (*N* = 64)	N (%)
< 1 week	23 (35.9)
1 week	17 (26.6)
2 weeks	17 (26.6)
3 weeks	2 (3.1)
4 weeks	5 (7.8)
Overall, how helpful was the OBGYN bootcamp course in preparing you for residency (*N* = 64)	N (%)
Not at all helpful	3 (4.7)
Slightly helpful	21 (32.8)
Moderately helpful	21 (32.8)
Very helpful	11 (17.2)
Extremely helpful	8 (12.5)
As part of your TTR course did you complete APGO’s PrepForRes quiz? (*N* = 115)	N (%)
Yes	22 (19.1)
No	77 (67.0)
Unsure	16 (13.9)
Did you receive a “handover” summarizing your clinical competency in ACGME milestones based on assessments during the TTR course that you could share with your Program Director? (*N* = 65)	N (%)
Yes	2 (3.1)
No	53 (81.5)
Unsure	10 (15.4)

### Educational activities and their perceived helpfulness

**Fig. 3:**
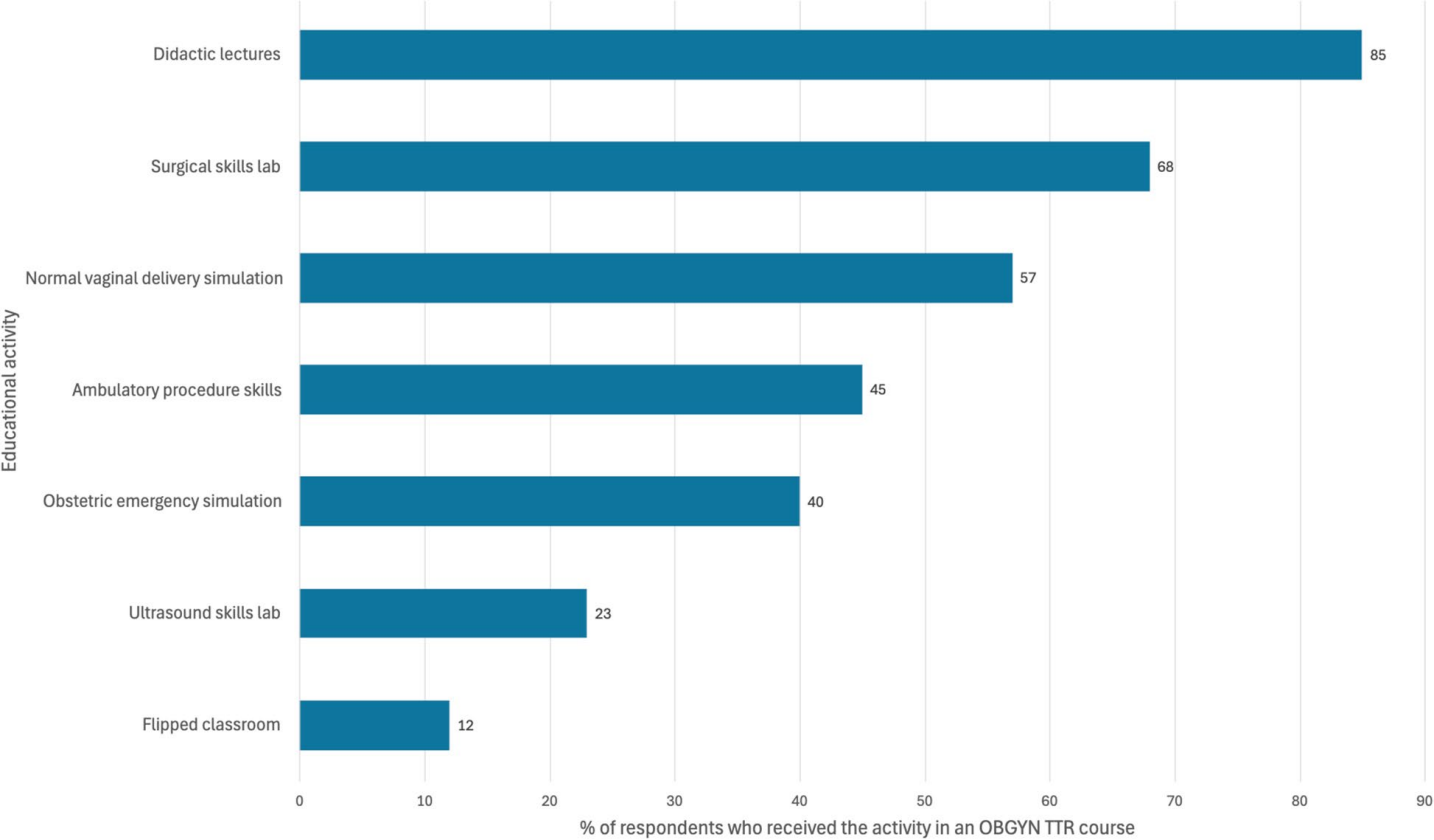
Educational activities utilized in OBGYN TTR Courses. Respondents who indicated that they received a an OBGYN TTR course were asked to select the educational activities employed in the course. (*n* = 66 respondents who received an OBGYN TTR course)

**Fig. 4:**
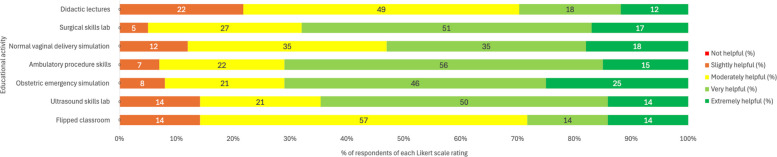
Perceived helpfulness of educational activities. Respondents who indicated that their OBGYN TTR course utilized each educational activity were asked to rate that activity on a Likert scale of helpfulness. (*n* = 66 respondents who received an OBGYN TTR course)

The incorporation of each educational activity is summarized in Fig. 3, and the perceived helpfulness of each is summarized in Fig. 4. Responses were included by both residents who had a stand-alone OBGYN TTR course and one nested within a general TTR course. The most frequently included activities were didactic lectures (85%), surgical skills labs (68%), and normal vaginal delivery simulation (57%). Overall, experiential learning was preferred over classroom learning. Each of the following experiential educational activities were perceived as “very helpful” or “extremely helpful” by more than half of respondents: ambulatory procedure skills (71%), obstetric emergency simulation (71%), surgical skills lab (68%), ultrasound skills lab (64%), and normal vaginal delivery simulation (53%). Of these most helpful modalities, only surgical skills lab and normal vaginal delivery simulation were utilized in at least half of reported courses.

### Educational topics and their perceived helpfulness

**Fig. 5:**
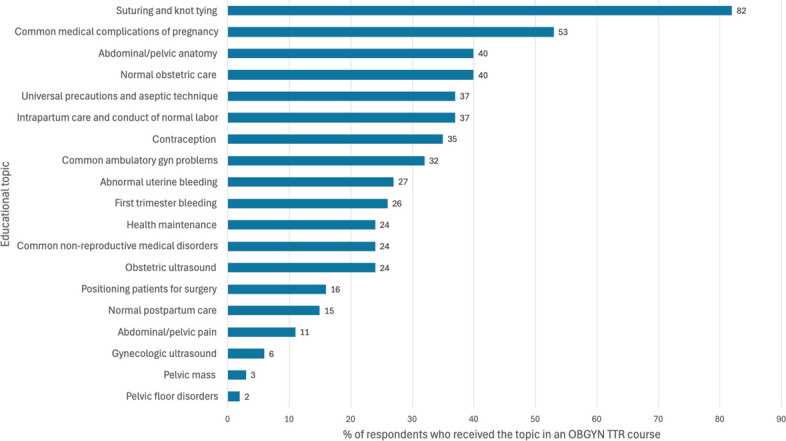
Educational topics taught in OBGYN TTR courses. Respondents who indicated that they received an OBGYN TTR course were asked to select the educational topics that were taught in the course. (*n* = 66 respondents who received an OBGYN TTR course)

**Fig. 6:**
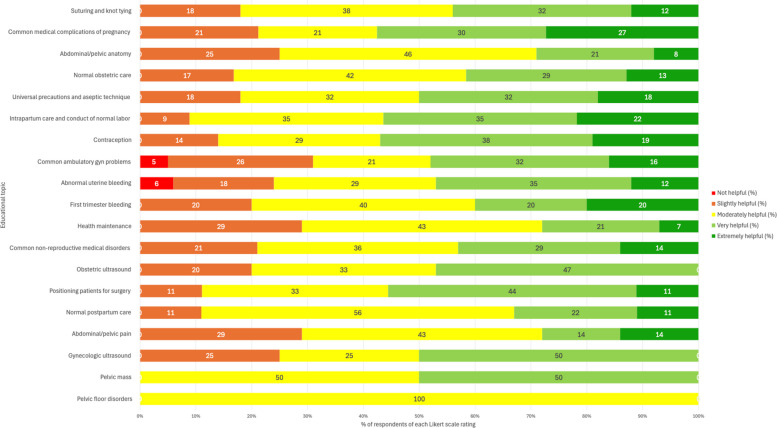
Perceived helpfulness of educational topics. Respondents who indicated that their OBGYN TTR course utilized each educational topic were asked to rate the teaching of that topic on a Likert scale of helpfulness. (*n* = 66 respondents who received an OBGYN TTR course)

**Fig. 7:**
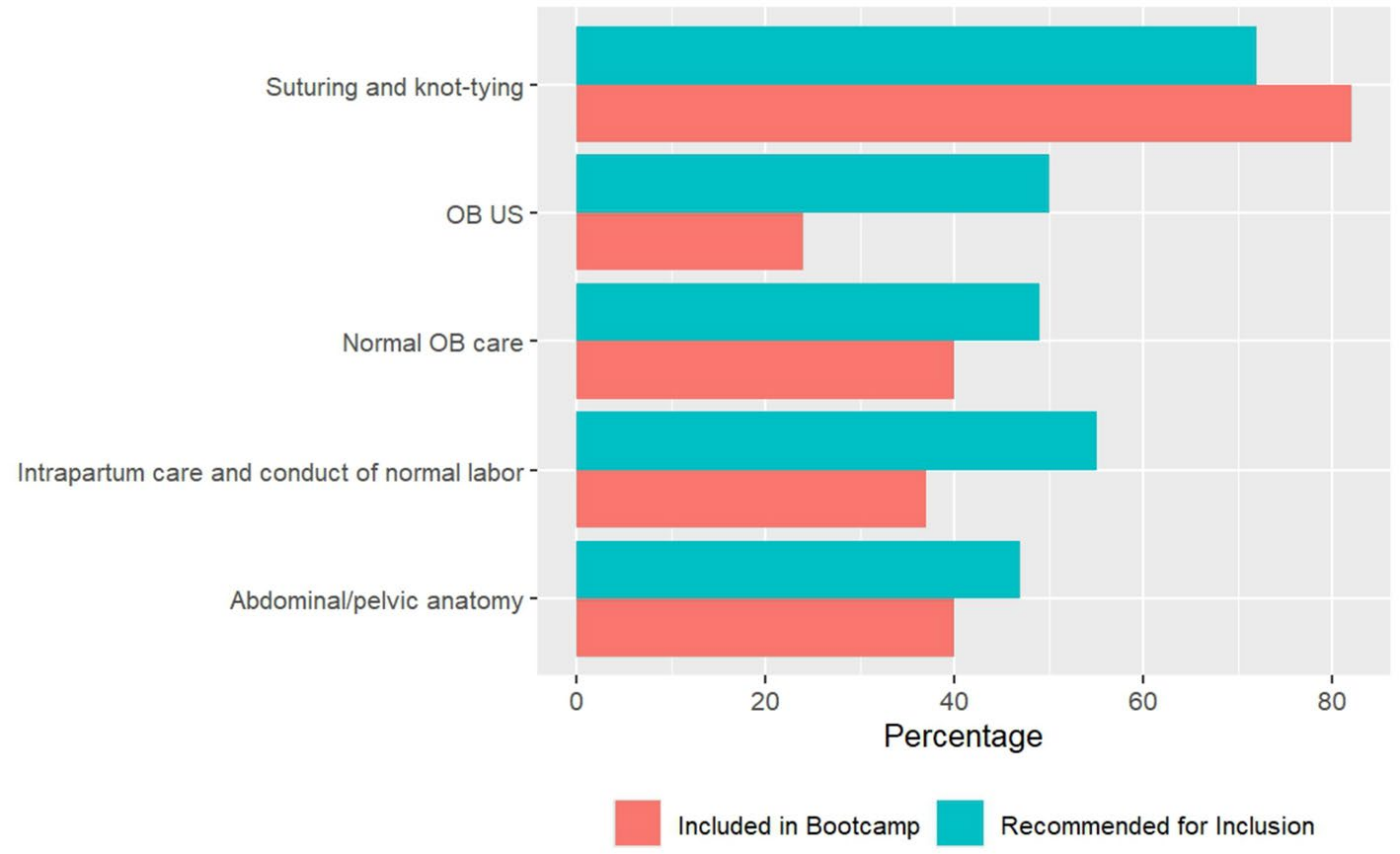
Comparison of the highest desired educational topics and their rate of inclusion. All respondents were asked to select 5 of the 19 ACGME Patient Care Milestone Level 1 mapped topics that would be most important to include in an OBGYN TTR “Bootcamp” course

The inclusion of ACGME Milestone Level 1 mapped OBGYN topics varied widely, as did respondents’ perception of their educational value. The inclusion of these topics in OBGYN TTR courses is summarized in Fig. 5 and their perceived helpfulness is summarized in Fig. 6. The most included topics were suturing and knot tying (82%) and medical complications of pregnancy (53%). Each of the following topics were perceived as “very helpful” or “extremely helpful” by more than half of respondents: common medical complications of pregnancy (57%), intrapartum care and conduct of normal labor (57%), contraception (57%), and positioning patients for surgery (55%). The topics considered most important to include in a TTR course were suturing and knot tying (79%), intrapartum care and conduct of normal labor (60%), obstetric ultrasound (55%), normal obstetric care (54%), and abdominal/pelvic anatomy (52%) (Fig. 7).

## Discussion

This study is the first to assess the breadth of curricular structure of OBGYN TTR courses and the perceived helpfulness of these courses from the perspective of OBGYN residents. Despite increasing national focus on incorporating specialty-specific preparatory courses in the MS4 year [6, 17], nearly half of respondents reported they did not receive OBGYN-specific training in a TTR course. A longer course duration (3–4 weeks) and stand-alone structure may be associated with higher perceived helpfulness, however, most respondents received OBGYN-specific training for a duration of two weeks or less within a general TTR course. In recognizing the time and structure constraints in many medical schools, it is important to prioritize the topics and learning styles respondents found to be most helpful for their training.

Residents reported higher approval of experiential learning over didactic learning. The activities with the highest perceived helpfulness included ambulatory, surgical, and ultrasound skills labs and simulations of normal vaginal delivery and obstetric emergency. Of these, only surgical skills lab and normal vaginal delivery simulation were included in more than half of courses reported. Didactic lectures were the most common modality and were largely considered moderately helpful. It should be noted that while experiential learning activities were perceived to be of higher value, they also require greater resources and time compared to didactic learning.

There is a large overlap between the topics respondents prioritized as most important to include in an OBGYN TTR course and those topics that are most frequently included. Among these are suturing and knot tying, normal obstetric care, intrapartum care and conduct of normal labor, and abdominal/pelvic anatomy. One exception was obstetric ultrasound, which was a highly valued topic but was only covered in 24% of the surveyed TTR courses. A previous study surveying program directors and interns identified ultrasound skills as a notable deficit among incoming residents [4]. Pilot curriculums of OBGYN TTR courses include obstetric ultrasound teaching through didactic lectures and simulation training [7, 18].

Additionally, while abdominal/pelvic anatomy is both highly desired by residents and one of the most frequently included topics, satisfaction of the teaching is lower when compared to other commonly taught topics. While the modalities being used to teach anatomy in such courses are not clear from this study, increased focus on experiential learning for this topic may increase its perceived helpfulness. Strategies that have been shown to improve resident knowledge in pelvic anatomy include clay and other 3D pelvic models, virtual reality, and cadaver dissection [19–22].

Most survey respondents had not taken APGO’s PrepForRes quiz nor had received a standardized handover from their medical school. Therefore, utilization of these objective assessments is an area for growth in the transition from undergraduate to graduate medical education [11, 23]. Morgan et al. published a perspective that detailed the ideal components of a handover resulting from a discussion at the 2018American Medical Association’s Accelerating Change in Medical Education consortium meeting and pilot studies completed at five medical schools [23]. This perspective determined that an ideal handover is a standardized form that details a medical student’s competency across ACGME-milestones. The assessment should actively involve the learner in identifying gaps of knowledge and creating an individualized learning plan with a coach or advisor. Ultimately, the handover should be provided to the learner’s residency program director at the conclusion of medical school with the goal of improving residency preparation. An OBGYN TTR course that focuses on the same ACGME-milestones as the both the PrepForRes quiz and competency handovers is an opportunity for increased utilization of these assessment tools and communication of the learner’s needs.

In general, most respondents did not find their TTR course to be significantly helpful, with only 30% indicating that they found the course “very” or “extremely” helpful. One reason residents are not completely content may be due to the discrepancy between learning modalities utilized and those with the most favorable reviews. However, the overly mediocre rating calls into question whether a TTR course is the appropriate place to invest increased time and money. Alternatives to TTR courses include the #ObGynInternChallenge, an SMS-distributed curriculum for self-directed learning [24], and intern bootcamps organized by residency programs in the 1–2 weeks prior to the beginning of residency [4]. These resources have distinct advantages and disadvantages. The #ObGynInternChallenge is more standardized and cost-effective compared to TTR courses; however, it relies solely on online self-guided learning [24]. Intern bootcamps are delivered closer to the start of residency but may be subject to time constraints of moving to a new location and payment for the residents’ time. Due to the benefits and limitations of each of these resources, we argue that they can and should be used concordantly to help supplement one another. Offering a TTR course focused on high-value topics using hands-on learning modalities and self-assessments may allow learners to identify pertinent knowledge gaps so they can utilize these additional resources with a targeted, focused approach.

The strengths of this study include the representation of residents from all years of training, a wide range of medical schools, and a broad geographic distribution. The survey generated responses representing graduates of 73 of the 193 (38%) U.S. medical schools from every U.S. census region. In addition, the study provides insight into both the content and structure of OBGYN TTR courses, as well as insight into the perceived educational value from the perspective of residents.

The study also has several limitations. There was a low response rate (21.9%), especially among residents in their final year of training, and only slightly more than half of respondents had received an OBGYN TTR course. Response rate may be improved in future studies by timing survey deployment around resident availability such as didactic or administrative blocks and with increased reminders through multiple channels (e.g. text and email). The low response rate raises concern regarding representativeness of the sample due to nonresponse and volunteer bias. Additionally, the small subgroup of respondents (*n* = 7) who received a 3–4 week TTR course, may be influenced by effort-related bias with a higher overall helpfulness rating than their counterparts. There was also a limitation of representativeness with regards to program type, as there were no strictly community-based programs included. Analysis was not modified to account for multiple submissions from the same medical school and TTR course, and there was no mechanism applied to avoid duplicate submissions which decrease data independence.

Information about TTR courses was obtained from graduates rather than course directors, which may introduce recall bias especially from respondents further along in training. On the other hand, the timing of the survey being deployed early in the residency year without a minimum experience inclusion criterion may have skewed the perception of current interns who have not had exposure to all rotations. Future studies can consider surveying PGY-1–2 residents regarding TTR experiences and PGY-3–4 residents regarding content recommendations.

Additionally, the study focuses on residents’ perception of helpfulness rather than day-one readiness. More objective measures such as skills assessment, knowledge testing, clinical outcomes, and self-confidence could indicate effectiveness of TTR courses. Respondents were not surveyed on some demographic information such as sex or gender; thus, it is possible that the study fails to recognize biases that may exist in competency perception among OBGYN residents [20]. Further studies can assess the performance of residents on standardized assessments such as APGO’s PrepforRes quiz and competency handovers to determine efficacy of TTR courses. It would be beneficial to assess which educational activities and modalities are being employed for each topic to evaluate a correlation among perceived value of the topic and its delivery.

Our findings support consideration of a standardized 2–4 week OBGYN TTR course with emphasis on experiential learning. Topics that should be prioritized include suturing and knot tying, normal obstetric care, intrapartum care and conduct of normal labor, abdominal/pelvic anatomy, and obstetric ultrasound. Other topics that should also be considered due to high satisfaction ratings include common medical complications of pregnancy, contraception, universal precautions and aseptic technique, and positioning patients for surgery.

## Conclusions

Overall, the results of this study indicate that OBGYN TTR courses are inconsistently implemented and vary in their structure, content, and perception among residents. Currently, OBGYN TTR courses are largely focused on the topics that residents value but fail to utilize many learning modalities residents perceive as the most helpful. Areas for improvement include increasing the number of medical schools that provide OBGYN TTR courses, prioritizing skills labs and simulations over didactics, and utilizing competency-based medical education assessments of incoming residents. Future research should assess the impact of standardized OBGYN TTR curricula on objective measures of intern readiness and performance.

## Supplementary Material

Supplementary Material 1.

## Data Availability

The datasets used and/or analyzed during the current study are available from the corresponding author on reasonable request.

## Abbreviations

TTR: Transition to Residency
U.S.: United States
OBGYN: Obstetrics and Gynecology
PGY: Postgraduate year
UGRC: Undergraduate Medical Education-Graduate Medical Education Review Committee
ACGME: Accreditation Council for Graduate Medical Education
CBME: Competency-based medical education
APGO: Association of Professors of Gynecology and Obstetrics
LCME: Liaison Committee for Medical Education
MD: Doctor of Medicine
DO: Doctor of Osteopathic Medicine
ME: Maine
NH: New Hampshire
VT: Vermont
MA: Massachusetts
RI: Rhode Island
CT: Connecticut
NY: New York
NJ: New Jersey
PA: Pennsylvania
OH: Ohio
MI: Michigan
IN: Indiana
IL: Illinois
WI: Wisconsin
MN: Minnesota
ND: North Dakota
SD: South Dakota
IA: Iowa
NE: Nebraska
MO: Missouri
KS: Kansas
DE: Delaware
MD: Maryland
DC: District of Columbia
WV: West Virginia
VA: Virginia
NC: North Carolina
SC: South Carolina
GA: Georgia
FL: Florida
KY: Kentucky
TN: Tennessee
AL: Alabama
MS: Mississippi
AR: Arkansas
OK: Oklahoma
LA: Louisiana
TX: Texas
MT: Montana
ID: Idaho
WY: Wyoming
CO: Colorado
UT: Utah
NV: Nevada
NM: New Mexico
AZ: Arizona
AK: Alaska
WA: Washington
OR: Oregon
CA: California
HI: Hawaii
