# Supplementary material for: Current practices in obstetrics and gynecology transition to residency courses: graduates’ perspectives on activities, topics, and overall educational benefit
Source: BMC Med Educ. 2026 Jun 12;26:1330. doi: 10.1186/s12909-026-08977-3 (PMC13483147; doi:10.1186/s12909-026-08977-3)
Supplement: Supplementary file 1 [file 12909_2026_8977_MOESM1_ESM.docx]

| APPENDIX A: OBGYN Transition to Residency Survey | |
| --- | --- |
| Q1. | Indicate your current position:   - PGY1 - PGY2 - PGY3 - PGY4 |
| Q2. | What medical school did you attend? |
| Q3. | Did you complete an OBGYN Transition to Residency “bootcamp” at the end of your fourth year of medical school?  *This does NOT include a sub-internship or regular 4^th^ year elective*   - Yes – I took a stand-alone OBGYN bootcamp elective - Yes – OBGYN content was integrated into a general transition to residency bootcamp - No – I did not receive OBGYN specific residency preparatory training |
| *Q4. | How long was the OBGYN-specific bootcamp training?   - < 1 week - 1 week - 2 weeks - 3 weeks - 4 weeks |
| *Q5. | Overall, how helpful was the OBGYN bootcamp course in preparing you for residency?   - Not at all helpful - Slightly helpful - Moderately helpful - Very helpful - Extremely helpful |
| Q6. | As part of your Transition to Residency course did you complete APGO’s PrepForRes quiz?   - Yes - No - Unsure |
| *Q7. | Did you receive a “handover” that you could share with your program director?  *A handover is an evaluation summarizing your clinical competency in ACGME milestones based on assessments during your Transition to Residency course*   - Yes - No - Unsure |
| *Q8. | Select all topics that were taught in your OBGYN bootcamp:   - Suturing and knot tying - Obstetric ultrasound - Gynecologic ultrasound - Abdominal/pelvic anatomy - Universal precautions and aseptic technique - Positioning patients for surgery - Normal obstetric care - Common medical complications of pregnancy - First trimester bleeding - Intrapartum care and conduct of normal labor - Normal postpartum care - Common ambulatory gynecology problems - Contraception - Abdominal/pelvic pain - Abnormal uterine bleeding - Pelvic mass - Pelvic floor disorders - Health maintenance - Common non-reproductive medical disorders (i.e. HTN, obesity, depression, osteoporosis) - None of the above |
| **Q9-27. | How helpful was [topic] content in preparing you for residency?   - Not at all helpful - Slightly helpful - Moderately helpful - Very helpful - Extremely helpful |
| *Q28. | Which of the following teaching modalities were employed in the OBGYN Transition to Residency course or breakout session?   - Didactic lectures - Flipped classroom (student-led didactics) - Simulation of normal vaginal delivery - Simulation of obstetric emergency - Ambulatory procedure skills lab (Endometrial biopsy, IUD insertion, etc.) - Surgical skills lab (laparoscopy, suturing, patient positioning, etc.) - Ultrasound skills lab - None of the above |
| ***Q29-35. | How helpful was [educational activity] in preparing you for residency?   - Not at all helpful - Slightly helpful - Moderately helpful - Very helpful - Extremely helpful |
| Q36. | Reflecting on your first months, which of the following content do you believe would be the most important to include in an OBGYN Transition to Residency course (select up to 5)   - Suturing and knot tying - Obstetric ultrasound - Gynecologic ultrasound - Abdominal/pelvic anatomy - Universal precautions and aseptic technique - Positioning patients for surgery - Normal obstetric care - Common medical complications of pregnancy - First trimester bleeding - Intrapartum care and conduct of normal labor - Normal postpartum care - Common ambulatory gynecology problems - Contraception - Abdominal/pelvic pain - Abnormal uterine bleeding - Pelvic mass - Pelvic floor disorders - Health maintenance - Common non-reproductive medical disorders (i.e. HTN, obesity, depression, osteoporosis) |

*Question prompted by Q3 selection of “yes - I took a stand-alone OBGYN bootcamp elective” or “Yes - OBGYN content was integrated into a general transition to residency bootcamp”

**Questions prompted by Q8 selected topics

***Questions prompted by Q28 selected educational activities

| APPENDIX B: ACGME Patient Care Milestones Level 1 for OBGYN residents | |
| --- | --- |
| *Patient Care Topic* | ***Milestone Level 1*** |
| Antepartum Care and Complications of Pregnancy | Demonstrates basic knowledge of normal obstetric care and common medical complications seen in pregnancy |
| First-Trimester Bleeding | Formulates a differential diagnosis for patients with first-trimester bleeding |
| Care of Patients in Intrapartum Period | Demonstrates basic knowledge of routine/uncomplicated intrapartum obstetrical care, including conduct of normal labor  Performs basic obstetric skills (e.g. identification of fetal lie, interpretation of fetal heart monitoring, and tocodynamometry) |
| Care of Patients in the Postpartum and Interconception Period | Provides postpartum and interconception care for women with uncomplicated pregnancies |
| Obstetric Technical Skills | Demonstrates basic surgical principles, including use of universal precautions and aseptic technique |
| Critically Ill Obstetric Patients and Obstetric Emergencies | Recognizes risk factors that can lead to critical illness in an obstetric patient  Recognizes risk factors for obstetric emergencies |
| Peri-Procedural care | Identifies alterations in normal physiology and anatomy |
| Endoscopic Procedures (Hysteroscopy and Cystoscopy) | Prepares patient and equipment for endoscopic procedures (e.g., lithotomy positioning, assemble endoscope) |
| Laparoscopic Procedures | Demonstrates basic skills (e.g., positioning, draping, docking, undocking) |
| Vulvar-Vaginal Procedures | Demonstrates basic skills (e.g., positioning, knot tying, suturing) |
| Open Surgical Procedures (excludes Caesarean section) | Demonstrates basic skills (e.g., positioning, knot tying, suturing) |
| Family Planning | Demonstrates ability to find reputable, evidence-based information on contraception choices |
| Ambulatory Gynecology and Office-Based Procedures | Takes a focused patient history for common ambulatory gynecologic problems |
| Consultations | Performs consultation, with guidance |

| APPENDIX C: OBGYN residency programs invited to participate in the study | | | |
| --- | --- | --- | --- |
| OBGYN residency program | **No. of residents** | **Institutional setting** | **Geographic region** |
| Abington Memorial Hospital | 24 | Community-based, university affiliated | Middle Atlantic |
| Beth Israel Deaconess Medical Center | 24 | University-based | New England |
| Carolinas Medical Center | 24 | Community-based, university affiliated | South Atlantic |
| Hackensack Meridian Health | 14 | University-based | Middle Atlantic |
| Henry Ford Providence Hospital | 19 | Community-based, university affiliated | East North Central |
| MaineHealth | 17 | Community-based, university affiliated | New England |
| Marshall University School of Medicine | 16 | Community-based, university affiliated | South Atlantic |
| The MetroHealth System / Case Western Reserve University | 20 | University-based | East North Central |
| New York Presbyterian Hospital (Columbia Campus) | 24 | University-based | Middle Atlantic |
| Ohio State University / Mt. Carmel Hospital | 44 | University-based | East North Central |
| Oregon Health & Sciences University | 39 | University-based | Pacific |
| Rutgers Health / Robert Wood Johnson Medical School | 20 | University-based | Middle Atlantic |
| Summa Health System / NEOMED | 24 | Community-based, university affiliated | East North Central |
| Tower Health / Reading Hospital | 20 | Community-based, university affiliated | Middle Atlantic |
| University of California Davis Health | 34 | University-based | Pacific |
| University of California Los Angeles David Geffen School of Medicine / UCLA Medical Center | 37 | University-based | Pacific |
| University of Colorado | 38 | University-based | Mountain |
| University of Florida | 19 | University-based | South Atlantic |
| University of Tennessee College of Medicine at Chattanooga | 15 | University-based | East South Central |
| Wake Forest University Baptist Medical Center | 28 | University-based | South Atlantic |
| Washington University/B-JH/SLCH Consortium | 35 | University-based | West North Central |
| Westchester Medical Center | 17 | University-based | Middle Atlantic |
